# Congenital Pouch Colon: A Preliminary Report from Pakistan

**Published:** 2012-07-01

**Authors:** Bilal Mirza, Sarfraz Ahmad, Afzal Sheikh

**Affiliations:** Department of Pediatric Surgery, The Children’s Hospital and the Institute of Child Health Lahore, Pakistan.

**Keywords:** Imperforate anus, congenital pouch colon, urogenital anomalies

## Abstract

Background: Congenital pouch colon (CPC) is a rare entity in patients of anorectal malformations (ARM) requiring special consideration as to the management. This study is aimed at evaluating the presentation, management, and the outcome of initial surgery in patients with CPC.

Materials and Methods: This retrospective study was conducted in the department of Pediatric Surgery of our institute during May 2007- May 2010. The Information about the demography, clinical features, investigations, management, and the outcome of initial surgery was retrieved and analyzed.

Results: There were 21 patients of CPC managed during the study period. Sixteen (76%) were males and five (24%) females (M:F 3.2:1). Mean age of presentation was 4.8 days with a range of 12 hours to 45 days. In 18 (85.7%) patients, CPC was found with high ARMs, whereas, in 3 (14.3%) patients it was associated with low ARMs. Imperforate anus with moderate to massive abdominal distension was the presentation in 16 (76%) patients. Abdominal radiographs helped in preoperative diagnosis in 8 patients. Two patients had pneumoperitoneum on abdominal radiographs. At operation, type I CPC was found in 9 (42.8%) patients, type II in 5 (23.8%), type III in 2 (9.5%) patients, and type IV CPC in 5 (23.8%) patients. In 11 (52.4%) patients, pouch was emptied and retained with proximal enterostomy. In 7 (33.3%) patients, end enterostomy with pouch excision was done. In two patients, a window colostomy was formed. In one patient, pouch was disconnected from the normal bowel and Hartmann’s pouch with end ileostomy was formed. There were 2 (9.5%) deaths in our series.

Conclusion: CPC is a rare malformation. Massive abdominal distension with imperforate anus is the common presentation. Optimum management can reduce the morbidity and mortality.

## INTRODUCTION

Congenital pouch colon is a variant of ARM, characterized by replacement of a variable length of normal colon by a pouch like structure associated with short length of the total colon along with fistulous communication with the genitourinary system. Interestingly, the entity has a special predilection for the northern areas of India and most of the literature regarding its embryology, etiology, anatomy, and management has been reported from the same region [1]. Sporadic cases or case series have been documented from rest of the world [2].

In Pakistan, a term “football colon” is attributed to complete congenital pouch colon. The condition is equally prevalent in Pakistan; however, no published literature on case series could be retrieved in spite of extensive search. Herein, we present our experience of managing the index entity in a tertiary care centre of Pakistan. The objective of the study was to identify various presentations, management provided, and the outcome of these patients in our hospital.

## MATERIALS AND METHODS

This is a retrospective review of patients of CPC, managed in the department of Pediatric Surgery, The Children’s Hospital and the Insti-tute of Child Health Lahore, Pakistan; during May 2007 through May 2010. The medical rec-ord of these patients was reviewed for demo-graphic information, clinical features, investiga-tions performed, operative notes, postoperative events, and the outcome of initial surgery. For descriptive purpose, CPC was classified into 4 types as per Rao et al classification (Table 1) [4].

**Figure:**
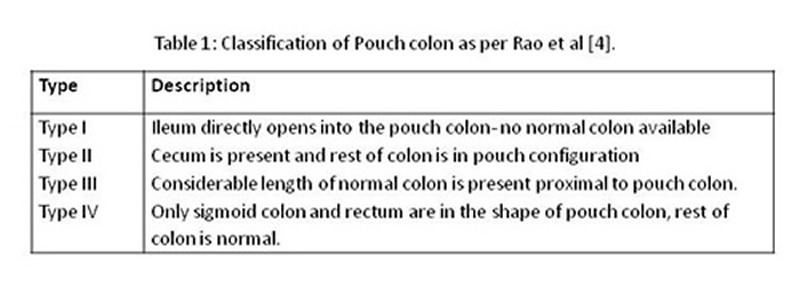
Table 1: Classification of Pouch Colon as per Rao et al[4]

## RESULTS

Demography: Of the 312 cases of ARM, 21 (6.73%) had associated pouch colon, male: female ratio being 3.2:1 (16 males and 5 females). The age of initial presentation ranged between 12 hours and 45 days with a mean of 4.8 days (SD ±9.8). The mean weight at presentation was 2.3 kg (SD±0.43) with a range of 1.8kg to 3.8kg.

Presentation: Nine patients presented with imperforate anus and abdominal distension. Table 2 describes clinical presentation of the study group. Of 21 patients, 18 (85.7%) were associated with high variety ARM and 3 (14.3%) with low ARM.

**Figure:**
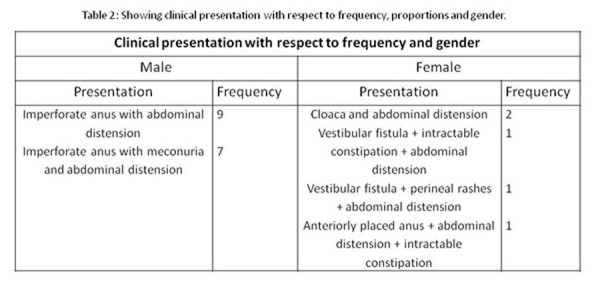
Table 2: Showing Clinical Presentation with respect to frequency, proportions and gender

Investigations: In 8 (38%) patients abdominal radiograph (erect and invertogram) delineated typical findings consistent with the diagnosis of CPC (Fig.1). Air vesicogram and pneumoperi-toneum was noted on radiographs in two patients each. Ultrasound was diagnostic of CPC in 5 patients only (23.8%).

**Figure:**
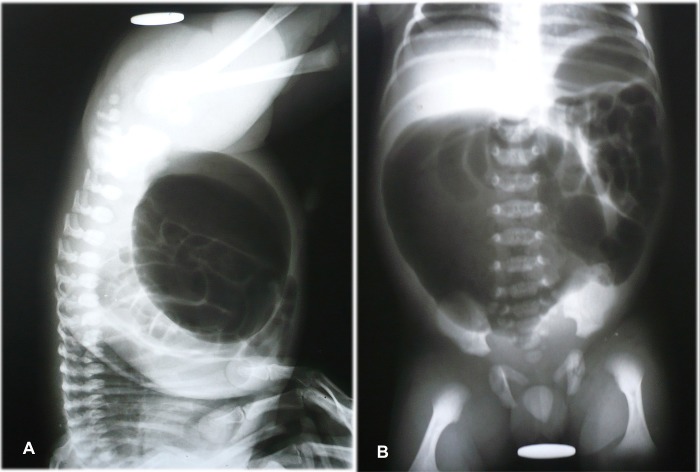
Figure 1: (A) Abdominal radiograph (invertogram) showing a large loop of bowel filled with meconium and air, occupying more than half of the abdominal cavity. (B) Erect radiograph of the same patient.

Operation: All the 21 patients underwent preliminary operations. Eight (38%) patients underwent exploratory laparotomy through a right supra-umbilical transverse incision. They all belonged to high ARM and were diagnosed preoperatively. In rest of the patients, preoperative diagnosis could not be made.

In 13 (62%) patients, CPC was diagnosed during colostomy formation. In 3 of these 13 patients, cecum was found in the left iliac region while constructing colostomy for ARM (Fig. 2,3); In another patient with colostomy for cloaca, type IV CPC was found on re-exploration for massive mucosal prolapse.

**Figure:**
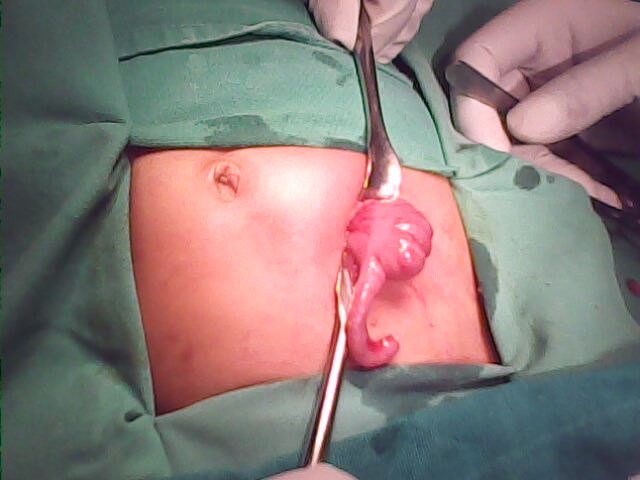
Figure 2: Cecum and appendix lying at abnormal position, observed during colostomy formation in a patient of ARM that later proved to have type IV CPC.

**Figure:**
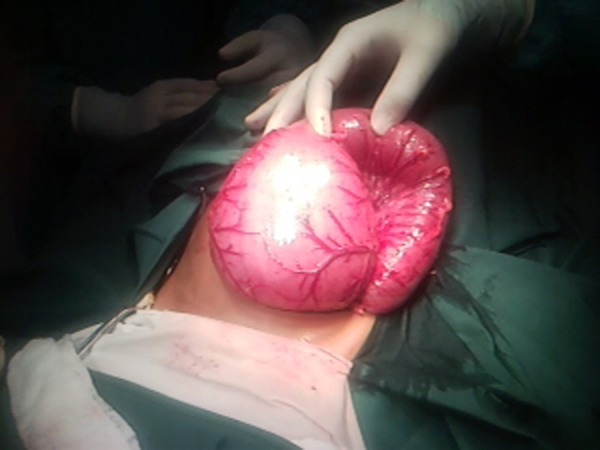
Figure 3: Pouch colon in another patient in whom cecum and appendix were observed in left iliac region.

In 9 patients, ileum directly opened into the pouch colon (Type I CPC). Various types of CPC encountered in the study population are tabulated here (Table 3). In two patients with type I CPC, there was perforation of the pouches at lower end of CPC (Fig.4,5).

**Figure:**
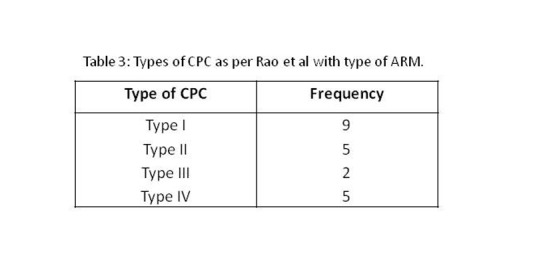
Table 3: Types of CPC as per Rao et al with type of ARM

**Figure:**
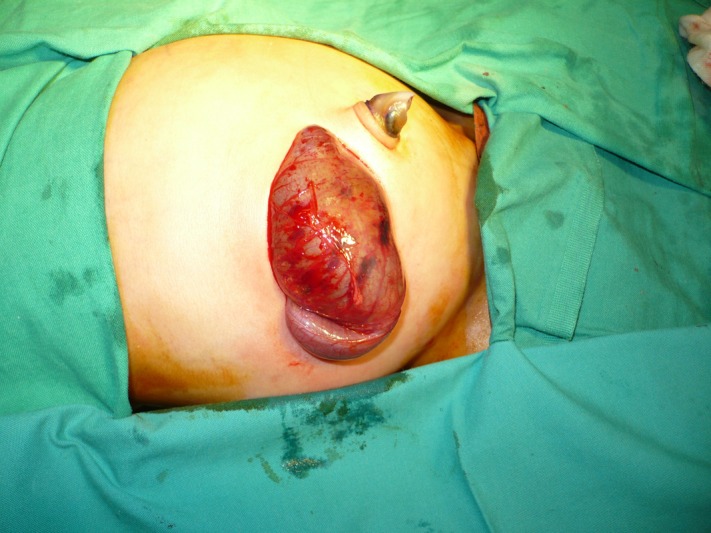
Figure 4: Operative view of Type I CPC; the ileum is directly opening into the CPC from right side.

**Figure:**
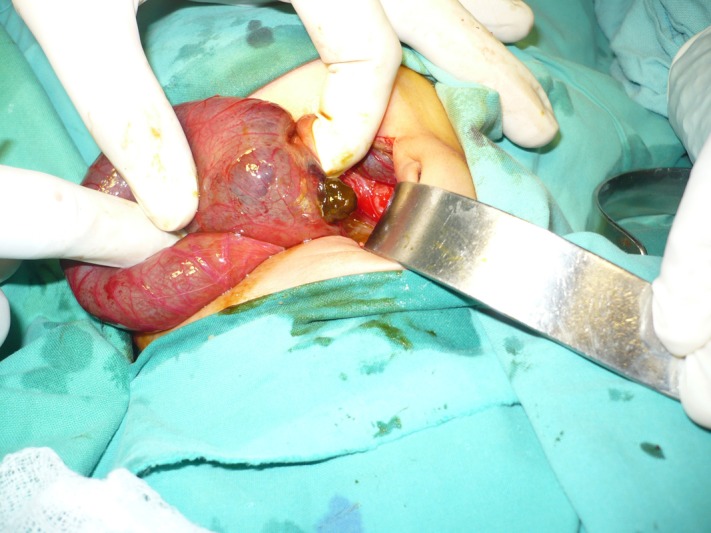
Figure 5: Operative view of a Type I CPC which was perforated at one side.

In all the male patients there was a fistula with the posterior wall of urinary bladder (Fig. 6), whereas in female patients the fistula was with the cloaca in 2, and with vestibule in 2, and in one patient the pouch was continuous with anteriorly placed anus.

**Figure:**
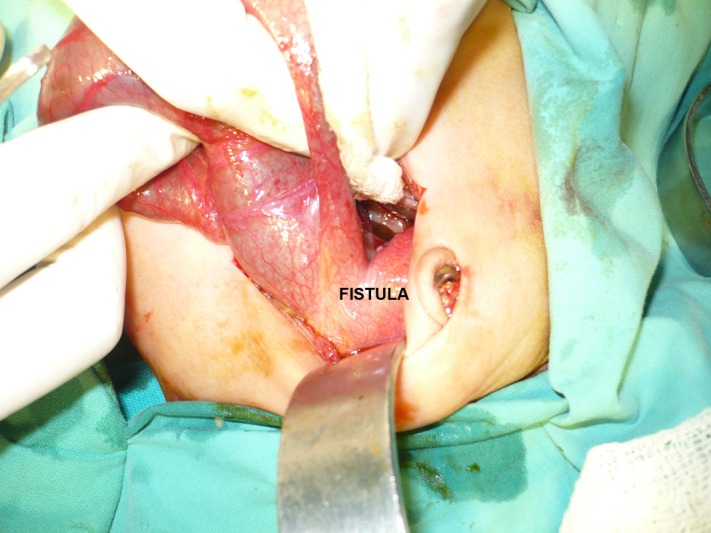
Figure 6: Operative view of a long and wide fistula with the posterior wall of urinary bladder.

In 4 of 9 patients with type I CPC, the pouch was emptied and retained with proximal ileostomy. In 3/9 patients with type I CPC the pouch was excised with end ileostomy. In 1 critical patient with type I pouch colon, the pouch was exteriorized as window colostomy. In one patient with type I CPC, the pouch was disconnected from the normal bowel and Hartmann’s pouch with end ileostomy was formed. The surgical treatment in the rest of study group is tabulated as under (Table 4).

**Figure:**
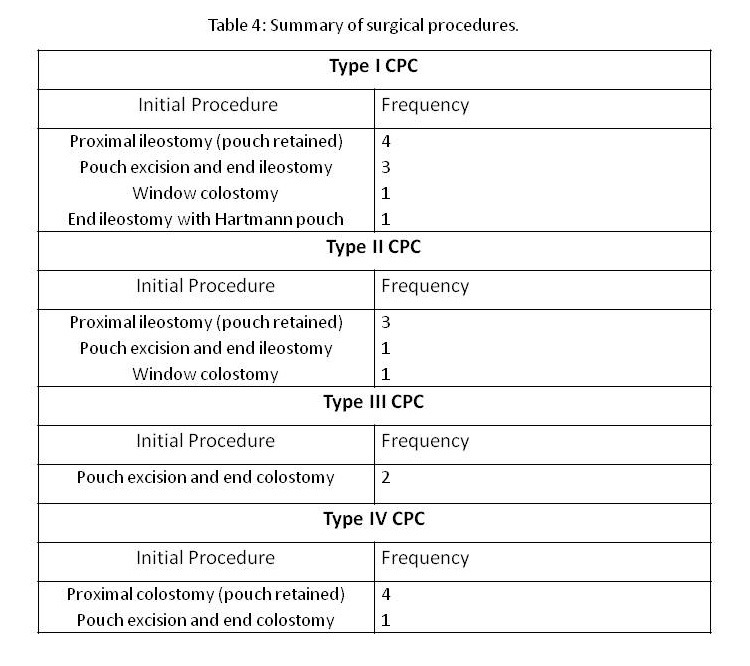
Table 4: Summary of Surgical Procedures

Associated Anomalies: Associated anomalies were mainly from genitourinary and gastrointestinal tract. Malrotation was observed in 5 patients, hydroureter in 2, and left renal agenesis in 1 patient. Table 5 describes the associated anomalies encountered in our series.

**Figure:**
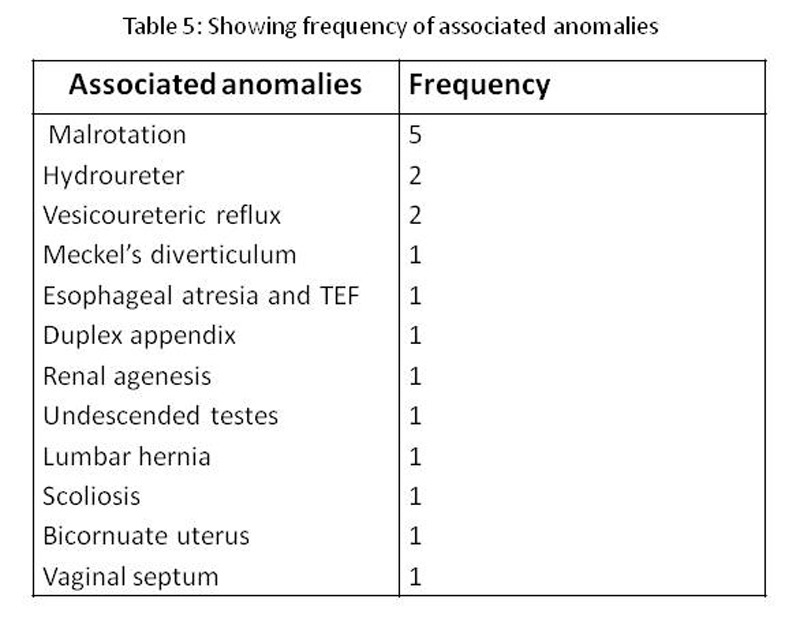
Table 5: Showing Frequency of associated anomalies

Complications: Postoperative complications encountered during initial surgery were wound infection in 4, mild wound dehiscence in 2, and enterostomy prolapse in 3 patients. The mortality in our series was 9.5% (2 patients); both the patients were critically sick since the presentation and one patient had associated esophageal atresia and tracheoesophageal fistula. 

## DISCUSSION

The incidence of CPC in our series was 6.73% of all the cases of ARM. The reported incidence of CPC in other series from India lies between 5-18% of all the cases of ARM [1-4]. The incidence of CPC in the western world is less than 1%. The exact incidence of this anomaly in Pakistan cannot be commented on as to scarce published data from this country. Similarly the sex distribution (M:F = 3.2:1)in our study was also in agreement with the reported male to female ratio (3-7:1) [1-7].

The presentation may be early or delayed depending upon gender, size of fistula and the type of the CPC. In males, massive abdominal distension with imperforate anus during first or second day of life is the usual early presentation, however in case of a wide fistula the patient may be decompressed and present with mild abdominal distension and muconuria. The presentation in females is usually delayed to their early infancy with intractable constipation, recurrent attacks of enterocolitis and persistent abdominal distension. The delayed presentation in females correlates with the type of ARM; the patients with associated cloaca usually present early as compared to vestibular fistula or other low ARM, where they are deliberately deferred to some later age for definitive surgery [1,5-8]. Two out of 5 female subjects with CPC in our series had presented early with cloaca. Of the other 3 patients, 2 presented with intractable constipation and one patient with perineal rashes due to recurrent attacks of enterocolitis and soiling of fecal matter. All the male patients presented early.

Preoperative diagnosis of CPC can be easily made with the help of radiographs (erect and invertogram). A large loop of bowel filled with air and meconium, occupying more than half of the abdominal cavity, is usually diagnostic of CPC in a patient with ARM. Sometimes additionally air vesicogram may be evident on radiographs [9]. In our series abdominal radiographs were performed in 10 patients that showed typical features of CPC in 8 and pneumoperitonium in 2 patients. In male patients with visible meconuria, and in female patients with common channel, radiographs were not performed on account of the clinical features depicting their type of ARM and straightforward decision of colostomy.

In the index study, type I CPC is frequent variety which corroborates with the previously published series [4,10-12]. The recent studies however documented type IV CPC being more prevalent than Type I or II varieties. This can be attributed to an increased awareness of the entity as to its morphology, pathology, and management in the evolving years [1,2,8].

The fistula with the genitourinary tract is characteristic of the index entity. Fistulous communication in males occurs with the urinary tract most often with the urinary bladder [1,9,13] as in our series, and in females the site of fistula varies from perineum to the vestibule to the cloacal malformation [5-7].

A number of genitourinary and gastrointestinal anomalies have been associated with CPC. Most common anomalies are hydrouretro-nephrosis, vesicouretric reflux, renal dysplasia/agenesis, bicornuate uterus, hypospadias, followed by malrotation, and appendicular duplex or agenesis, and prune belly syndrome [1,14]. In our series, 11 patients had associated anomalies, the commonest being malrotation. In one female patient of type IV CPC (anteriorly placed anus) there was associated scoliosis and lumber hernia.

CPC can be managed as a primary one stage procedure or as staged procedure. Single stage procedure ideally consists of repair of fistula, excision of the pouch, and primary abdomino-perineal-PSARP/pull through [1,12]. Multi-staged procedures are variable and range from merely window colostomy to complete excision of CPC, repair of the recto-vesical fistula, and end enterostomy in the first stage followed by pull through of the normal bowel with/without covering enterostomy as a second stage procedure. Initially single stage procedures were not considered safe but recent reports emerged with good results. Pouch colon patch over the pulled through segment is also advocated [1,12, 16-19]. Window colostomy is indicated in critical patients, but it is often associated with high morbidity and mortality. The problems with window colostomy are massive mucosal prolapse, urinary tract infections, incomplete emptying of the CPC resulting in persistent abdominal distension etc. [1]. The outcome in CPC has improved with mortality reduced from >40% to less than 10% [1,5].

## Conclusion

To summarize, CPC is rare malformation in patients of ARM though the incidence in the Asian subcontinent is higher than in the western world. Imperforate anus with massive abdominal distension is a common presentation of this anomaly. An erect plain abdominal radiograph is diagnostic and should be done in all patients of ARM with abdominal distension. If one encounters cecum and appendix in the left lower quadrant while colostomy formation for ARM, it is mandatory to look for CPC.

## Footnotes

**Source of Support:** None

**Conflict of Interest:** None

